# Insights from engraftable immunodeficient mouse models of hyperinsulinaemia

**DOI:** 10.1038/s41598-017-00443-x

**Published:** 2017-03-28

**Authors:** Michelle L. Maugham, Patrick B. Thomas, Gabrielle J. Crisp, Lisa K. Philp, Esha T. Shah, Adrian C. Herington, Chen Chen, Laura S. Gregory, Colleen C. Nelson, Inge Seim, Penny L. Jeffery, Lisa K. Chopin

**Affiliations:** 1 Ghrelin Research Group, Translational Research Institute, Institute of Health and Biomedical Innovation, and School of Biomedical Sciences, Queensland University of Technology, Brisbane, Queensland, Australia; 2 Australian Prostate Cancer Research Centre - Queensland, Institute of Health and Biomedical Innovation, Queensland University of Technology, Princess Alexandra Hospital, Translational Research Institute, Brisbane, Queensland, Australia; 3 Comparative and Endocrine Biology Laboratory, Translational Research Institute, Institute of Health and Biomedical Innovation, Queensland University of Technology, Brisbane, Queensland, Australia; 4 Skeletal Biology and Forensic Anthropology Research Laboratory, Cancer Program, School of Biomedical Sciences, Translational Research Institute (TRI), Institute of Health and Biomedical Innovation, Queensland University of Technology, Brisbane, Queensland, Australia; 5 School of Biomedical Sciences, University of Queensland, St Lucia, Brisbane, Queensland, Australia

## Abstract

Hyperinsulinaemia, obesity and dyslipidaemia are independent and collective risk factors for many cancers. Here, the long-term effects of a 23% Western high-fat diet (HFD) in two immunodeficient mouse strains (NOD/SCID and *Rag1* ^−/−^) suitable for engraftment with human-derived tissue xenografts, and the effect of diet-induced hyperinsulinaemia on human prostate cancer cell line xenograft growth, were investigated. *Rag1* ^−/−^and NOD/SCID HFD-fed mice demonstrated diet-induced impairments in glucose tolerance at 16 and 23 weeks post weaning. *Rag1* ^−/−^ mice developed significantly higher fasting insulin levels (2.16 ± 1.01 ng/ml, *P* = 0.01) and increased insulin resistance (6.70 ± 1.68 HOMA-IR, *P* = 0.01) compared to low-fat chow-fed mice (0.71 ± 0.12 ng/ml and 2.91 ± 0.42 HOMA-IR). This was not observed in the NOD/SCID strain. Hepatic steatosis was more extensive in *Rag1* ^−/−^ HFD-fed mice compared to NOD/SCID mice. Intramyocellular lipid storage was increased in *Rag1* ^−/−^ HFD-fed mice, but not in NOD/SCID mice. In *Rag1* ^−/−^ HFD-fed mice, LNCaP xenograft tumours grew more rapidly compared to low-fat chow-fed mice. This is the first characterisation of the metabolic effects of long-term Western HFD in two mouse strains suitable for xenograft studies. We conclude that *Rag1* ^−/−^ mice are an appropriate and novel xenograft model for studying the relationship between cancer and hyperinsulinaemia.

## Introduction

Metabolic syndrome encompasses obesity and several related conditions, including insulin resistance, dyslipidaemia, inflammation, and cardiovascular disease^1^. While these comorbidities are intrinsically linked^2–4^, some are independent risk factors for cancer^5–7^. Many components of metabolic syndrome promote gastrointestinal, endometrial, breast, and prostate cancers and increase the likelihood of lethal, higher-grade disease^8–11^. Despite growing recognition of the strong link between metabolic disturbance and cancer progression, there are few engraftable rodent models of human cancer suitable for investigating this association. A number of mouse models of obesity, hyperinsulinaemia, and hyperglycaemia arose from spontaneous genetic mutations^12^. This includes leptin-deficient (*Lep°* ^*b/*^ *°* ^*b*^)^13^, leptin receptor-deficient (*LepR* ^*db/db*^)^14^, and agouti yellow obese mice (*A* ^*y*^ */a*)^15^. Diet-induced models of metabolic syndrome have also been developed. Consuming a high-fat diet (HFD) induces obesity with insulin resistance in rats^16, 17^, and a high-fat, high-simple carbohydrate diet models human type 2 diabetes mellitus (T2DM) in C57BL/6 J mice^18^.

As inflammation is involved in the development of metabolic syndrome^19^, most studies of diet-induced obesity employ immunocompetent mice, such as the C57BL/6 J strain^12^ which develops insulin insensitivity as a result of chronic inflammation when fed a 42 or 60% HFD^20, 21^. Some immunodeficient strains, including severe combined immunodeficiency (SCID), non-obese diabetic/severe combined immunodeficiency (NOD/SCID), and NOD/SCIDIL2Rγ (NSG) mice, may be resistant to developing HFD-induced metabolic syndrome, due to a lack of adaptive immunity and defective innate immunity^22, 23^. For example, NOD/SCID mice, which are immunocompromised due to a spontaneous mutation in *Prkdc* ^24^, develop streptozotocin-induced, but not diet-induced hyperglycaemia^25^. Streptozotocin-induced pancreatic insulitis, a well-established model of type I diabetes mellitus, destroys pancreatic β-cell function, thereby abrogating insulin secretion. This model does not recapitulate the complex interplay between hyperinsulinaemia, β-cell stress and apoptosis, and insulin resistance phenotype of T2DM, however^26^. In contrast, mice with inactivating mutations in the genes encoding the RAG1 or RAG2 proteins, *Rag1* and *Rag2*, are susceptible to diet-induced hyperglycaemia^20, 21, 27^. RAG1 and RAG2 are involved in activating the recombination of T-cell receptor molecules and immunoglobulin genes, and a null mutation of either of these genes results in adaptive immunity deficiencies, with an absence of mature B and T lymphocytes^28^. *Rag1* ^−/−^ mice backcrossed onto a C57BL/6 J genetic background rapidly develop insulin resistance one week after initiation of 60% HFD feeding^20^. Additionally, when fed a 42% fat diet, *Rag1* ^−/−^ mice on a C57BL/6 J background gain more weight and fat mass than wild-type C57BL/6 J mice. Both *Rag1* ^−/−^and wild-type C57BL/6 J mice exhibit glucose and insulin intolerance after 10 weeks on this diet^21^. SCID, NOD/SCID and NSG mice are often employed in xenograft studies, as the rate of xenograft establishment is high for many cancer cell lines^29^. These strains are resistant to diet-induced insulin resistance^30^, and therefore, are not suitable for studying tumour biology associated with hyperinsulinaemia.

The aim of our study was to develop a diet-induced model of hyperinsulinaemia in immunocompromised mice suitable for cancer xenograft studies. In order to confirm previous studies^22, 23^, and to determine if *Rag1* ^−/−^ mice provide a better engraftable model for metabolic dysfunction, we compared the effect of a normal, low-fat chow and a Western, moderate-fat diet (23% fat diet, 45% digestible energy from fat) on weight gain, glucose tolerance, hormone levels and adiposity in *Rag1* ^−/−^ mice (on a C57BL/6 J background) and NOD/SCID mice. This study is the first to show that a Western HFD results in more significant diet-induced metabolic dysfunction in *Rag1* ^−/−^ mice compared to NOD/SCID HFD-fed mice. Furthermore, a pilot study demonstrates that LNCaP human prostate cancer cell line xenografts grow more rapidly in *Rag1* ^−/−^ mice fed a Western HFD than tumours in control *Rag1* ^−/−^ mice fed a low-fat diet. The *Rag1* ^−/−^ mouse, therefore, provides a novel model for studying tumour biology associated with diet-induced hyperinsulinaemia.

## Results

### Effect of a high-fat diet on NOD/SCID and *Rag1*^−/−^ mice

#### Glucose tolerance, insulin levels and HOMA analysis

**Figure 1:**
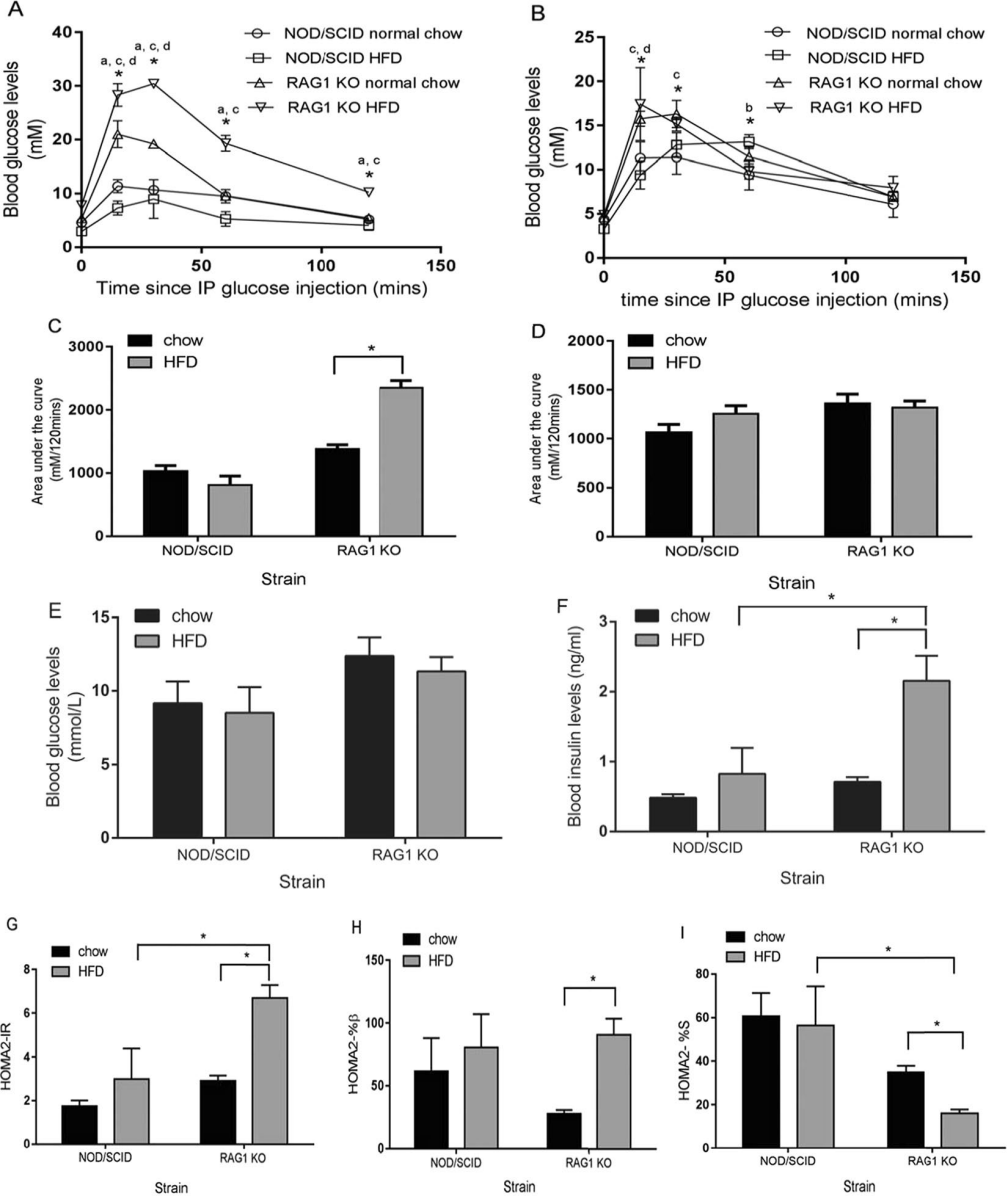
The effects of high-fat diet (HFD) or low-fat chow on blood glucose and insulin levels, insulin resistance, and insulin sensitivity in *Rag1* ^−/−^ and NOD/SCID mice. (**A**) Glucose tolerance (IPGTT) is impaired 16 weeks post-weaning in 23% high-fat diet (HFD) fed *Rag1* ^−/−^ mice (n = 8) compared to *Rag1* ^−/−^ mice fed low-fat chow (n = 4), and NOD/SCID mice fed HFD (n = 8), or low-fat chow (n = 4). (**B**) Glucose tolerance (IPGTT) is improved 23 weeks after diet initiation in *Rag1* ^−/−^ HFD-fed mice, while glucose tolerance is impaired in NOD/SCID mice fed HFD. (**C**) Blood glucose levels (measured over 120 minutes of IPGTT and expressed as area under the curve) in *Rag1* ^−/−^ and NOD/SCID mice fed low-fat chow or HFD at 16 weeks. (**D**) Blood glucose levels (measured over 120 minutes of IPGTT and expressed as area under the curve) at 23 weeks post diet initiation. (**E**) Fasting blood glucose levels measured at 28 weeks post-weaning. (**F**) Fasting insulin levels (ELISA) measured 28 weeks after diet initiation are significantly higher in *Rag1* ^−/−^ HFD-fed mice compared to *Rag1* ^−/−^ mice fed low-fat chow and NOD/SCID HFD-fed mice. (**G**) Insulin resistance, (Homeostatic model assessment for insulin resistance, HOMA-IR) is significantly higher in *Rag1* ^−/−^ HFD-fed mice. (**H**) Steady-state β-cell function determined by Homeostatic model assessment for β-cell function (HOMA-β) is significantly higher in *Rag1* ^−/−^ HFD-fed mice. (**I**) Insulin sensitivity, estimated using Homeostatic model assessment for (HOMA-S), is significantly lower in *Rag1* ^−/−^ mice on a HFD. Mean + SEM. Two-way ANOVA and Tukey's multiple comparisons test *P ≤ 0.05. a = *Rag1* ^−/−^ HFD *vs*. *Rag1* ^−/−^ low-fat chow-fed mice, b = NOD/SCID HFD-fed *vs*. NOD/SCID low-fat chow-fed mice, c = *Rag1* ^−/−^ HFD *vs.* NOD/SCID HFD-fed mice, d = *Rag1* ^−/−^ low-fat chow *vs.* NOD/SCID low-fat chow-fed mice. All other data was tested for statistical significance using the Kruskal-Wallis and Mann-Whitney test. *P ≤ 0.05.

Intraperitoneal glucose tolerance tests (IPGTT) were conducted 16 and 23 weeks after diet initiation in *Rag1* ^−/−^ and NOD/SCID mice to determine the effect of HFD (23% fat) on glucose tolerance (Fig. 1A,B). Blood glucose levels were elevated (AUC analysis, 2345 ± 116 mM/120 mins) in *Rag1* ^−/−^ mice fed a HFD for 16 weeks, compared to *Rag1* ^−/−^ mice fed low-fat chow (1380 ± 67.61 mM/120 mins, *P* = 0.001), and higher blood glucose levels were observed in HFD-fed mice at all time points (Fig. 1A) (*P *≤ 0.05). After 23 weeks, glucose tolerance improved in the HFD-fed *Rag1* ^−/−^ mice (AUC 1319 mM/120 ± 66.89 mins at 23 weeks post-weaning, relative to the 16-week time point) (Fig. 1C,D). In low-fat chow-fed *Rag1* ^−/−^ mice, however, glucose tolerance remained stable at 16 (AUC 1380 mM/120 mins ± 67.61) and 23 weeks post-weaning (AUC 1362 mM/120 mins ± 94.18) (Fig. 1C,D).

Glucose tolerance in HFD-fed NOD/SCID mice was more impaired at 23 weeks (AUC 1256 mM/120 ± 81.0 mins) (Fig. 1D) than at 16 weeks (810 ± 143.5 mM/120 mins) (Fig. 1C). Glucose levels were elevated at the 30 and 60 min time points at 23 weeks (*P* = 0.042) (Fig. 1B). No significant differences in glucose tolerance were observed between 16 (1028 ± 89.5 mM/120 mins) and 23 weeks post-weaning (1064 ± 81.15 mM/120 mins) in NOD/SCID low-fat chow-fed mice (Fig. 1C,D).

In mice fed a high-fat diet, blood glucose levels were significantly lower in NOD/SCID mice compared to *Rag1* ^−/−^ mice (*P* = 0.026) at 16 weeks (Fig. 1A). Similarly, on low-fat chow diet, NOD/SCID mice had significantly lower blood glucose levels compared to *Rag1* ^−/−^ mice at the 15 and 30 minute GTT time points at 16 (*P* = 0.026) and 23 weeks (*P* = 0.026) (Fig. 1A,B). Blood glucose levels were significantly higher in *Rag1* ^−/−^ HFD-fed mice compared to NOD/SCID HFD-fed mice at the 15-minute GTT time point at 23 weeks (*P* = 0.026) (Fig. 1B).

Fasting blood glucose and insulin were determined at endpoint (28 weeks post-weaning) in order to estimate insulin resistance, insulin sensitivity and steady-state β-cell function using HOMA2^31, 32^. Fasting insulin levels were significantly higher in *Rag1* ^−/−^ mice fed HFD (2.16 ± 1.01 ng/ml) compared to low-fat chow-fed *Rag1* ^−/−^ (0.712 ± 0.116 ng/ml, *P* = 0.012), and compared to NOD/SCID HFD-fed mice (*P* = 0.028) (Fig. 1F). No significant difference was observed between NOD/SCID groups (Fig. 1F). HOMA insulin resistance (HOMA-IR) was significantly greater in *Rag1* ^−/−^ HFD-fed mice (6.70 ± 1.68) compared to low-fat chow-fed controls (2.91 ± 0.42, *P* = 0.012) (Fig. 1G), but not affected by diet in NOD/SCID mice. Higher levels of insulin resistance were observed in HFD-fed *Rag1* ^−/−^ mice compared to NOD/SCID HFD-fed mice (*P* = 0.032). Similarly, steady-state β-cell function (HOMA-β) (Fig. 1H) was significantly higher in *Rag1* ^−/−^ HFD-fed mice (90.7 ± 35.8) compared to low-fat chow-fed mice (27.87 ± 5.00) (*P* = 0.012). Insulin sensitivity (HOMA-S) was significantly lower in *Rag1* ^−/−^ HFD-fed mice (15.97 ± 4.88) compared to low-fat chow-fed *Rag1* ^−/−^ mice (34.90 ± 5.31, *P* = 0.012) and HFD-fed NOD/SCID mice (*P* = 0.032) (Fig. 1I). No significant difference was observed between NOD/SCID groups.

#### The effect of high-fat diet on body weight in NOD/SCID and *Rag1*^−/−^ mice

**Figure 2:**
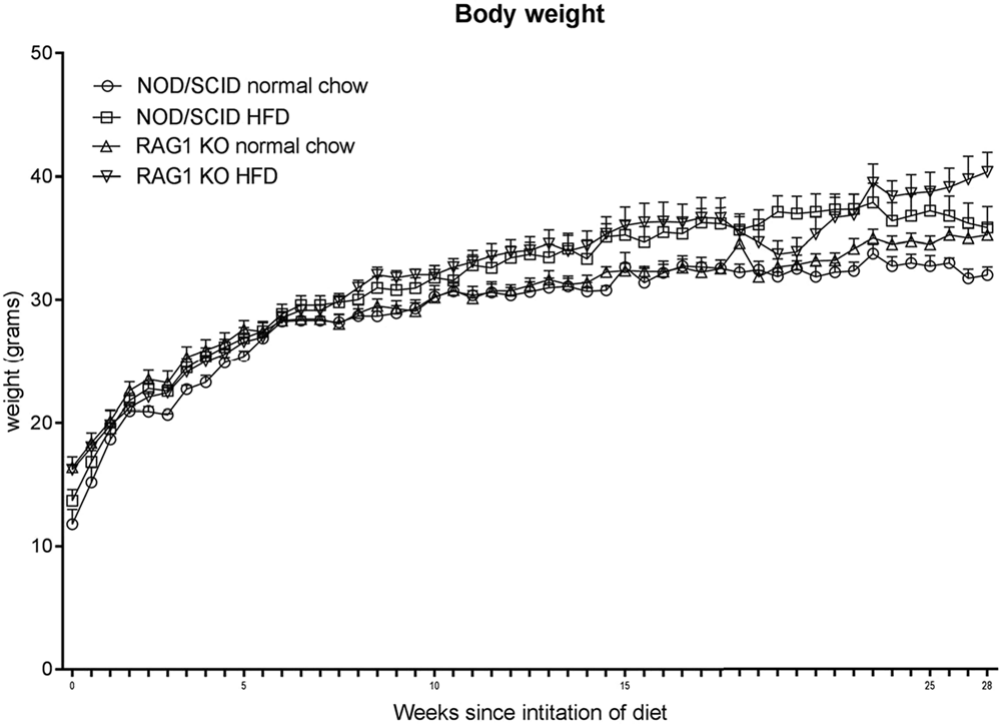
Mean body weight in NOD/SCID and *Rag1* ^−/−^ mice fed a high-fat diet (HFD) (23% fat, n = 8) compared to normal low-fat chow-fed control groups (n = 4). Mean + SEM.

*Rag1* ^−/−^ and NOD/SCID mice were fed HFD (23% fat) or low-fat diet from 3 weeks of age and the effect on bodyweight determined. *Rag1* ^−/−^ HFD-fed mice had higher bodyweights than low-fat chow-fed *Rag1* ^−/−^ mice from 2 weeks post weaning (Fig. 2). At endpoint, body weight was almost 20% higher in the HFD *Rag1* ^−/−^ group, however, these differences were not statistically significant (*P* = 0.0791) (Fig. 2). At endpoint, white adipose tissue was observed at the gross level during dissection in both HFD-fed mouse strains, however, brown adipose tissue (interscapular) was present in *Rag1* ^−/−^ HFD-fed mice only.

#### Histological analysis of adipose, skeletal muscle, hepatic and pancreatic tissue

**Figure 3:**
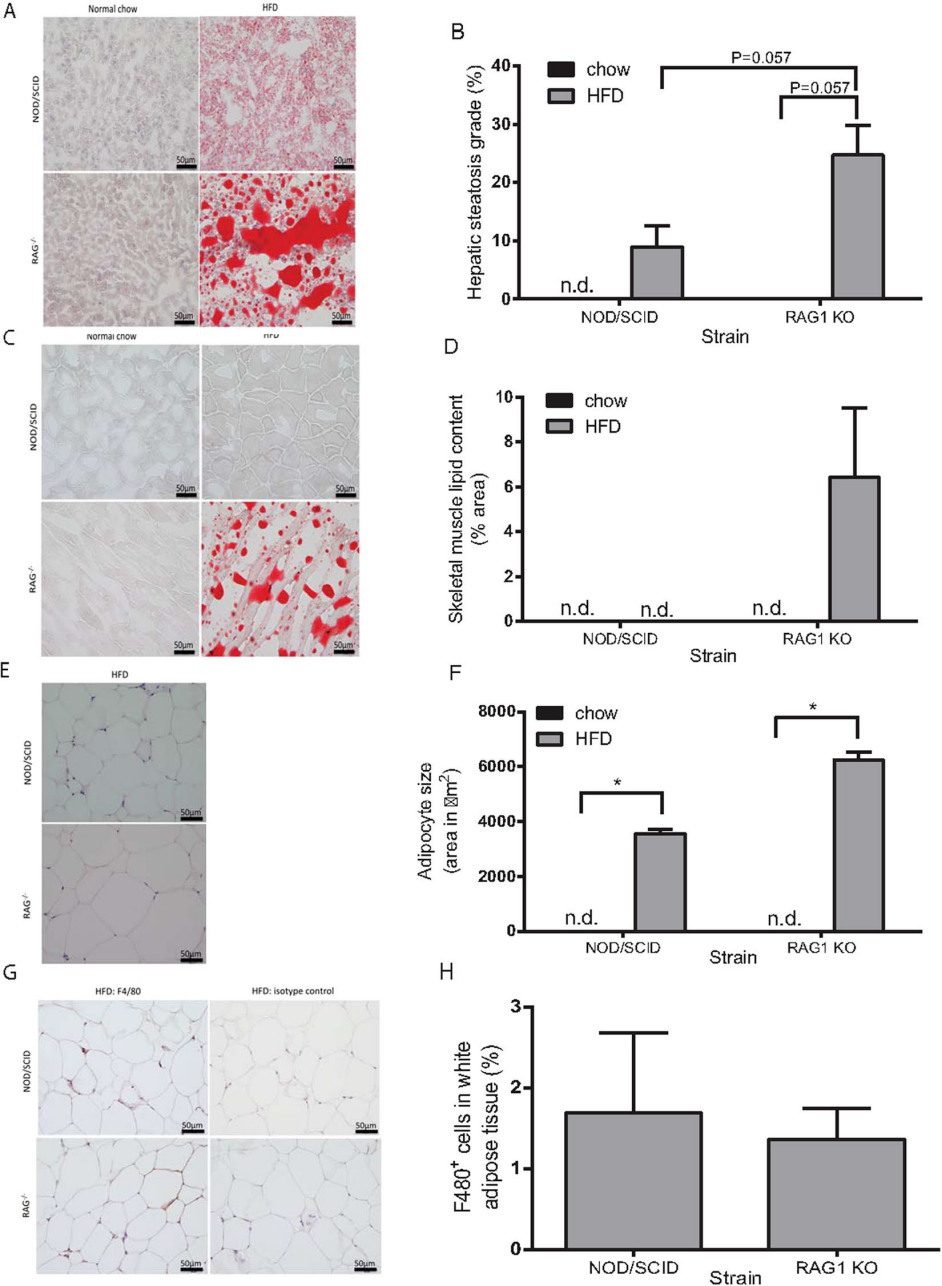
HFD increases lipid storage in *Rag1* ^−/−^ and NOD/SCID mice with more pronounced effects in *Rag1* ^−/−^ mice. (**A**) Oil-red-O stained liver histological sections demonstrate hepatic lipid in NOD/SCID and *Rag1* ^−/−^ mice fed HFD for 28 weeks. Hepatic lipid is absent in mice fed low-fat chow. (**B**) Hepatic steatosis (% oil-red-O stained hepatic adipocyte area) in *Rag1* ^−/−^ and NOD/SCID mice fed HFD. (**C**) Lipid accumulation in oil-red-O stained skeletal muscle in *Rag1* ^−/−^ mice fed HFD, but not in *Rag1* ^−/−^ normal chow-fed mice, or NOD/SCID mice. (**D**) Intramyocellular lipid content (% oil-red-O stained adipocyte area) in *Rag1* ^−/−^ mice fed HFD. (**E**) White adipose tissue deposits (haematoxylin and eosin) in HFD-fed NOD/SCID and *Rag1* ^−/−^ mice, are absent in mice fed low-fat chow. (**F**) Mean adipocyte size (expressed as area) is greater in *Rag1* ^−/−^ mice fed HFD compared to NOD/SCID HFD-fed mice. (**G**) F4/80 positive (brown) immunostaining of white adipose tissue deposits demonstrates macrophage infiltration in white adipose tissue in NOD/SCID and *Rag1* ^−/−^ mice fed HFD. (**H**) Percent of F4/80 positive stained cells in HFD-fed NOD/SCID and *Rag1* ^−/−^ mice white adipose tissue. Mean + SEM. Kruskal-Wallis and Mann-Whitney test. *P ≤ 0.05. n.d. = not detectable. Scale bar = 50 µm.

Skeletal muscle and hepatic tissue were stained with oil-red-O (ORO) to determine the degree of lipid content (percent area). HFD-fed *Rag1* ^−/−^ mice had greater hepatic lipid content (24.7 ± 10.1%, n = 4) than low-fat chow-fed *Rag1* ^−/−^ mice (0.051 ± 0.089%, n = 3, *P* = 0.057) (Fig. 3A,B). Lipid was not detectable in the liver of low-fat chow-fed NOD/SCID mice. NOD/SCID HFD-fed mice exhibited lower hepatic lipid content (8.92 ± 7.41%, n = 4) compared to HFD-fed *Rag1* ^−/−^mice (24.7 ± 10.1%, n = 4, *P* = 0.057) (Fig. 3A,B). Intramyocellular lipids were present in *Rag1* ^−/−^ mice fed HFD (6.42 ± 5.34%, n = 3), but not in low-fat chow-fed *Rag1* ^−/−^ mice (n = 3, *P* = 0.10), nor in NOD/SCID mice on either diet (n = 3) (Fig. 3C,D).

Epididymal fat pad white adipose tissue was not visible in low-fat chow-fed NOD/SCID, or *Rag1* ^−/−^ mice (n = 4) (Fig. 3E). Adipocytes were larger in HFD-fed *Rag1* ^−/−^ mice (6252 ± 583 µm^2^, n = 4) compared to HFD-fed NOD/SCID mice (3549 ± 315 µm^2^, n = 3, *P* = 0.057). F4/80 immunohistochemistry revealed the presence of infiltrating macrophages in white adipose tissue deposits in both NOD/SCID and *Rag1* ^−/−^ HFD-fed mice (Fig. 3G,H).

#### Growth of LNCaP subcutaneous xenografts is increased in hyperinsulinaemic, HFD-fed *Rag1*^−/−^ mice

**Figure 4:**
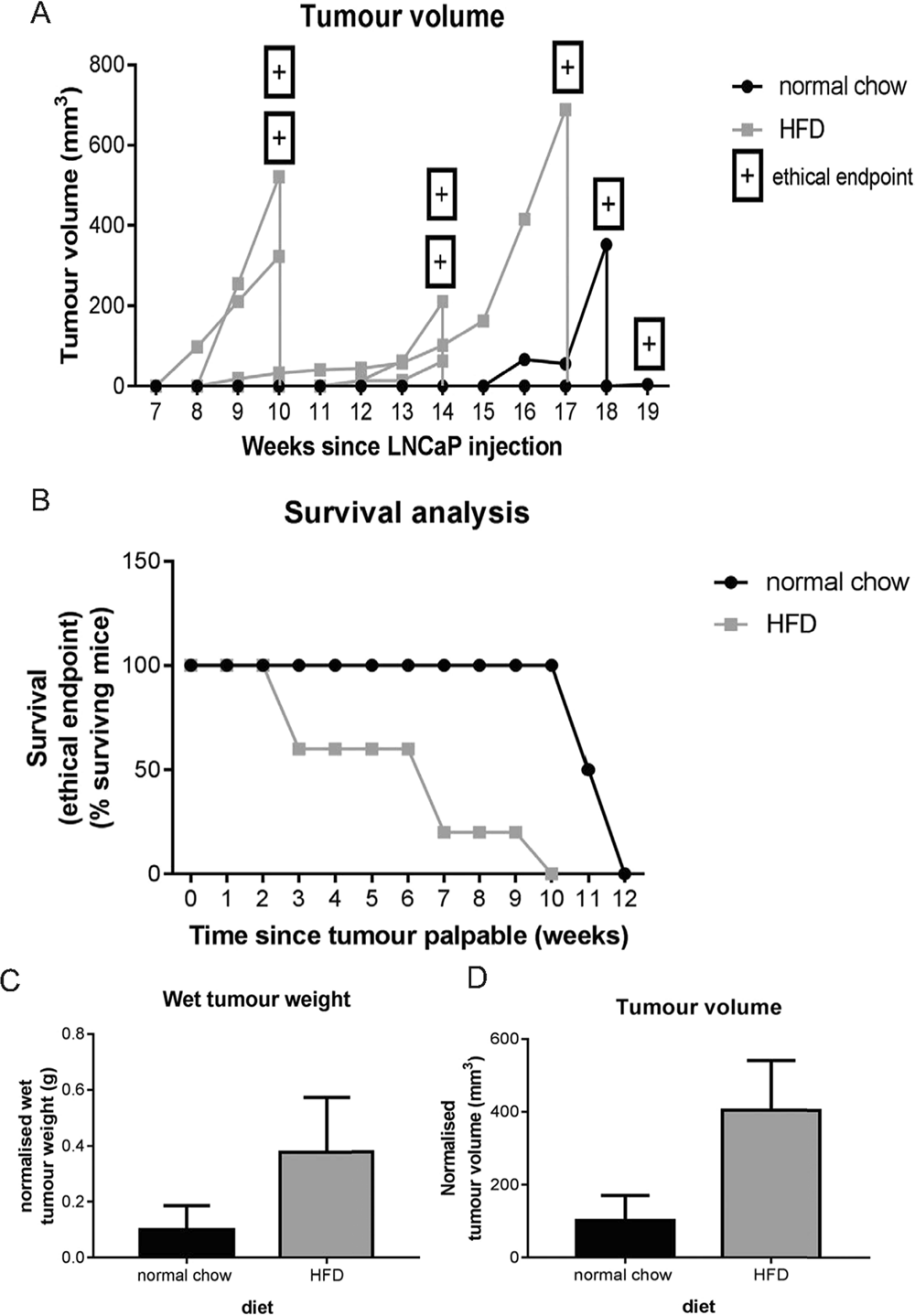
LNCaP subcutaneous xenografts grow more rapidly in HFD-fed *Rag1* ^−/−^ mice. (**A**) HFD-fed *Rag1* ^−/−^ mice (n = 5) develop larger and earlier tumours (shown as tumour volume) over time (weeks since subcutaneous xenograft implantation) compared to normal chow-fed mice (n = 2). *P* = *NS*. Wilcoxon matched-pairs signed rank test. (**B**) Survival to ethical endpoint (from time of palpable tumour in weeks) is significantly shorter in mice fed HFD. Log-rank test. (**C**) Wet tumour weight (normalised to time since xenograft injection) at endpoint is greater in HFD mice. *P* = *NS*. Mann-Whitney test. (**D**) Tumour volume (normalised to time since xenograft injection) at endpoint is greater in HFD-fed mice *P* = *NS*. Mann-Whitney test. Mean + SEM.

LNCaP xenograft tumours were palpable 8 weeks earlier in *Rag1* ^−/−^ HFD-fed mice than low-fat chow-fed *Rag1* ^−/−^ mice and tumours exhibited more rapid growth (Fig. 4A,C,D). Survival (to ethical endpoint) was significantly decreased in HFD-fed *Rag1* ^−/−^ mice (54.55 ± 10.82% surviving mice, n = 5) compared to low-fat chow-fed mice (88.46 ± 8.31% surviving mice, n = 2, *P* = 0.034) (Fig. 4B). Metabolic parameters, including fasting blood insulin levels, insulin resistance, and steady-state β-cell function at endpoint, were also measured in *Rag1* ^−/−^ mice fed HFD (see Supplementary Fig. S1), however, an increase in the group size is needed before statistically significant conclusions can be drawn.

## Discussion

The present study is the first to investigate the long-term (28 week) metabolic effects of a Western, 23% high-fat diet (HFD) on two immunodeficient mouse strains suitable for human xenograft implantation. We demonstrate that the metabolic effects of 23% HFD (46% energy from fat) are more pronounced in *Rag1* ^−/−^ compared to NOD/SCID mice, manifesting as higher fasting insulin levels, increased insulin resistance and steady-state β-cell function, lower insulin sensitivity, and increased adipose accumulation in skeletal muscle and liver tissue. The *Rag1* ^−/−^ strain is, therefore, a suitable immunodeficient mouse model for investigating HFD-induced hyperinsulinaemia. Furthermore, our pilot LNCaP mouse xenograft study revealed that a moderate HFD promotes tumour growth. Compared to low-fat chow-fed *Rag1* ^−/−^ mice, HFD-fed *Rag1* ^−/−^ mice developed tumours earlier, and exhibited increased tumour growth over time and decreased survival to ethical endpoint.

*Rag1* ^−/−^ mice in this study demonstrated impaired glucose tolerance within 16 weeks of HFD initiation. We surmise that this is likely to be a reflection of their predominantly C57BL/6 J genetic background (N6). It is well established that the C57BL/6 J strain is genetically predisposed to developing metabolic syndrome when fed a HFD^18, 33^. Previous studies, in which *Rag1* ^−/−^ and C57Bl/6 J mice were fed a Western diet (42.2% calories from milk fat and 42.8% calories from carbohydrate) for 11 weeks, demonstrated that these strains develop impaired glucose tolerance compared to low-fat diet fed mice, and no significant difference was observed between *Rag1* ^−/−^ and C57BL/6 J HFD-fed mice^21^. The *Rag1* ^−/−^ mouse bred onto a C57BL/6 J genetic background is, therefore, a useful model for studying the effects of insulin resistance, hyperinsulinaemia and, potentially, other components of the metabolic syndrome on cancer development and progression.

NOD/SCID and NSG mice are commonly used for xenograft studies due to their high tumour engraftment rate^34^, however, they are relatively resistant to diet-induced hyperinsulinaemia^22^ as they lack fully competent immune systems. *Rag1* ^−/−^ mice mice (on a BALB/c background) have been used for cancer allograft and xenograft studies for prostate cancer^35^. Similarly, *Rag2* ^−/−^ mice, which lack mature B and T lymphocytes, due to a mutation in the recombination-activating gene encoding the RAG2 protein^36^, have been employed to study endometrial cancer^37^ and oral squamous cell carcinoma^38^. Classically, *Rag1* ^−/−^ mice may not be considered as amenable to xenograft studies, as they retain some innate immune function, including moderate natural killer cell (NK) activity, which reduces engraftment rate and may distort the architecture of engrafted tumours^39^. However, as the development of insulin resistance is an inflammatory process^2^, the presence of some innate immunity may be advantageous for the study of HFD and cancer. Indeed, we reveal that engraftment of human prostate cancer cell line xenografts is possible in both HFD and chow-fed *Rag1* ^−/−^ mice, with tumours developing to a palpable size more rapidly in HFD-fed mice.

Impaired glucose and insulin tolerance have been reported to develop in *Rag1* ^−/−^ mice as early as 1 week after initiation of a 60% HFD^20^, however, this high-fat content is not representative of a human diet. The diet used in our study is closer to a Western human diet, with 46% energy from fat, (14.31% of which is saturated fat), 34% carbohydrate and 20% protein. A study by Liu and colleagues^21^ employed a comparable diet, with similar energy from fat (42.2% energy) but a higher carbohydrate (42.8%) and lower protein content (15%) than the diet used in our study. Studies where mice were fed 60% or 46% HFD^20, 21^ revealed similar diet-induced metabolic changes, including significantly impaired glucose tolerance in HFD-fed mice compared to chow-fed controls after 10–16 weeks on the diet^21^. Our study investigated chronic effects (28 weeks) of a high-fat diet, permitting the assessment of pancreatic β-cell function over time and demonstrating that increased β-cell activity compensates for hyperglycaemia, allowing a progression to hyperinsulinaemia. Our study is novel, as it compares the metabolic effects of HFD on two commonly-used xenograft hosts, *Rag1* ^−/−^ and NOD/SCID mice. Both strains lack a competent adaptive immune system, however, only the NOD/SCID mice lack competent innate immunity^22, 23^.

To the best of our knowledge, this is the first study of a Western HFD in *Rag1* ^−/−^ mice. In these mice, fasting blood glucose measurements at 28 weeks, and glucose tolerance at 23 weeks, improved compared to 16 weeks; possibly as a result of β-cell compensation and a significant increase in blood insulin levels. HOMA revealed significantly greater insulin resistance and steady-state β-cell function, and lower insulin sensitivity compared to low-fat chow-fed mice. This is likely to reflect the diabetogenic C57BL/6 J genetic background of *Rag1* ^−/−^ mice. Similarly, glucose tolerance improves with age in C57BL/6 J mice fed a normal chow diet as a result of age-related increases in islet size and pancreatic insulin content^40^. Our pilot LNCaP xenograft study (conducted in mice backcrossed with C57BL/6 mice for 10 generations) demonstrates that *Rag1* ^−/−^ mice fed a Western HFD display changes in metabolic parameters, however, greater sample size is needed to determine if these changes are statistically significant.

Although glucose tolerance in HFD-fed *Rag1* ^−/−^ mice improved with time, glucose tolerance in NOD/SCID mice became progressively impaired. NOD/SCID HFD-fed mice also developed symptoms of metabolic disturbance, including increased plasma insulin, insulin resistance, hepatic steatosis, and increased adipocyte size, however, these changes were more pronounced in HFD-fed *Rag1* ^−/−^ mice. Although present in *Rag1* ^−/−^ HFD-fed mice, intramyocellular lipid was not observed in NOD/SCID mice. Previous studies on the effect of 46% or 60% HFD in *Rag1* ^−/−^ mice investigated adipose tissue accumulation in subcutaneous, epididymal, mesenteric and perirenal depots, but lipid accumulation within metabolic organs crucial for energy homeostasis, such as the liver and skeletal musculature, were not measured^20, 21^. Increased hepatic steatosis and skeletal muscle lipid accumulation measured in HFD-fed *Rag1* ^−/−^ mice in our study is, thus, a novel finding.

Interestingly, *Rag1* ^−/−^ HFD mice were the only group observed to retain interscapular brown adipose tissue at endpoint. Brown adipose tissue has a high thermogenic capacity and plays a role in body weight control^41, 42^. This observation may suggest that *Rag1* ^−/−^ HFD-fed mice adapt to a high-fat diet, partly through increased brown adipose tissue mass and minimised weight gain, to maintain energy homeostasis. As the animal facility was maintained at 20–23 °C, and the murine thermoneutral range is 30–33 °C^43^, fat stores may have been depleted in low-fat chow-fed animals in an attempt to maintain body temperature^44^.

The immune system and inflammation have an integral role in the pathogenesis of obesity, type 2 diabetes mellitus (T2DM) and metabolic syndrome^19^, and the majority of metabolic studies have employed immunocompetent mice^12^. In this study we demonstrate that white adipose tissue was infiltrated with F4/80 positive macrophages in both NOD/SCID and *Rag1* ^−/−^ HFD-fed mice, while animals fed normal chow lacked visible adipose deposits. The infiltration of adipose tissue by macrophages in obesity is thought to play a critical role in mediating insulin resistance and the development of T2DM, triggering β-cell apoptosis and reducing the secretion of insulin^2, 45^. Our observation correlates with previous studies demonstrating significantly increased activated macrophage-related cytokine IL-12^46^ in the circulation of *Rag1* ^−/−^ HFD-fed mice compared to *Rag1* ^−/−^ fed a low-fat diet (16.7% energy from fat)^21^.

Our study demonstrates that a Western 23% HFD increases fat mass, reduces insulin tolerance, increases LNCaP human prostate cancer xenograft growth, and decreases survival to ethical endpoints in male *Rag1* ^−/−^ mice. Further studies are required, however, to determine if the *Rag1* ^−/−^ mouse is a useful model for investigating the interaction between HFD consumption and female cancers, particularly given the growing body of evidence describing gender-specific responses to HFD in C57BL/6 mice^47^. Specifically, male mice fed a HFD develop hyperinsulinaemia and low-grade systemic inflammation, whereas females do not^47^ - possibly due to the anti-inflammatory effects of oestrogen and expansion of regulatory T cells in female mice fed a HFD^48, 49^. Given the strong link between the development of endometrial and breast cancers and metabolic syndrome^10, 50^, the *Rag1* ^−/−^ model is likely to be useful for the further investigation of this association.

In conclusion, this is the first study to show that *Rag1* ^−/−^ mice fed a Western 23% HFD from weaning develop a number of symptoms associated with metabolic dysfunction, including hyperinsulinaemia, increased fasting insulin levels, insulin resistance, decreased insulin sensitivity, increased adiposity, hepatic steatosis and intramyocellular lipid accumulation. NOD/SCID mice fed the same diet developed some metabolic sequelae, however, these effects were more pronounced in *Rag1* ^−/−^ mice. Although further studies are required, this study demonstrates that a Western 23% HFD in *Rag1* ^−/−^ mice increases the growth rate of prostate cancer xenografts and significantly decreases survival to ethical endpoint compared to low-fat chow-fed mice. The *Rag1* ^−/−^ immunodeficient mouse is a promising mouse model for exploring the interaction between metabolic disturbances and the development and progression of cancers associated with symptoms of metabolic syndrome.

## Methods

### Hyperinsulinaemic mouse model and pilot xenograft study

To establish hyperinsulinaemia in immunocompromised mice, male 3-week-old NOD.CB17-*Prkdc* ^*scid*^/Arc (NOD/SCID) and recombination-activation gene deficient mice (B6.SVJ129-*Rag1* ^*tm1Bal*^/Arc; *Rag1* ^−/−^) (Jackson Laboratories; supplied by Animal Resource Centre, Murdoch, WA, Australia) were weaned onto an *ad libitum* diet of low-fat, normal chow (4.8% fat, 20% protein, 75.2% carbohydrate, 12152, Specialty Feeds, Glen Forrest, WA, Australia, http://www.specialtyfeeds.com), or Western, high-fat diet (23% fat, 46% digestible energy from fat, 20% energy from protein, 34% energy from carbohydrate, SF04–027, Specialty Feeds) (n = 4–8 per mouse strain and diet). *Rag1* ^−/−^ mice were backcrossed onto a C57BL/6 J background for six generations (N6; Animal Resources Centre). Mice were maintained on this diet for 28 weeks in total, with bodyweight monitored twice weekly. LNCaP human prostate cancer cell line xenograft studies were performed using *Rag1* ^−/−^ mice backcrossed onto a C57BL/6 J background for ten generations (N10; Animal Resources Centre). Mice were initiated on HFD, or low-fat normal chow at weaning (4 weeks of age), and injected with 2 × 10^6^ LNCaP cells in Dulbecco's Phosphate Buffered Saline (DPBS) (Thermo Fisher, Waltham, MA, USA) at a 1:1 ratio with growth factor reduced Matrigel (Sigma-Aldrich, St. Louis, MO, USA) in the subcutaneous tissue of the right flank at 6 weeks of age. Mice were maintained on HFD or low-fat normal chow, and body weight and tumour volume monitored weekly using calipers. For mice with LNCaP xenografts, experimental endpoint was determined by tumour volume (>1000 mm^3^), calculated using the equation ‘tumour volume = length × width^2^/2’, or if an ethical endpoint was reached (based on a combination of signs of stress including increased heart rate, inactivity, reduced interaction with cage mates, abnormal posture and/or >20% body weight loss as per ethical approval and the Australian Code). Xenograft volume was normalised for different durations to ethical endpoint after implantation using the equation ‘(xenograft volume/time since implantation) × 100’. Metabolic parameters (see Supplementary Fig. 1) were normalised for time since weaning using the equation ‘(original measurement/time) × 100’. Mice were housed under pathogen-free conditions in individually-ventilated cages, at a room temperature of 20–23 °C, with a 12 hour light-dark cycle. All methods were conducted in accordance with ethical guidelines and regulations. Animal ethics approval was granted from the University of Queensland and Queensland University of Technology animal ethics committees, and human ethics approval for cell line (LNCaP) use was granted from Queensland University of Technology Human Research Ethics Committee.

### Intraperitoneal glucose tolerance test

At 16 and 23 weeks after initiation of the diet, intraperitoneal (i.p.) glucose tolerance tests were performed (n = 4 mice per group) to determine effect of diet on glucose tolerance. Mice were fasted for 16 hours and baseline glucose levels measured in tail-tip blood with a One-touch Ultra blood glucose monitoring system and test strips (Accu-Chek Performa, Roche, Basel, Switzerland). Glucose (20% solution, 2 g/kg) was injected i.p. and blood glucose levels assessed at 15, 30, 60 and 120 minutes post injection. Fasting blood glucose was measured at the endpoint of the experiment (28 weeks post weaning). Surrogate indices of insulin resistance, insulin sensitivity and steady-state β-cell function were determined using the homeostatic model for assessment calculator (HOMA2)^51^, available from the Oxford Centre for Diabetes, Endocrinology and Metabolism^31^, using measured fasting glucose and insulin levels. HOMA analysis is an accepted surrogate for measuring insulin resistance in rodents^52^.

### Blood and tissue sample preparation

Blood for biochemical measurements was collected by terminal endpoint cardiac puncture. Tissues of interest (brown fat, epididymal fat pad, liver and skeletal muscle) were excised, frozen in Tissue-Tek O.C.T. embedding compound (VWR, Radnor, PA, USA), and stored at −80 °C or fixed in 4% paraformaldehyde for histological and immunohistochemical analysis.

### Hormone measurement

Fasting serum insulin was determined by ELISA (EMD Merck Millipore Group, Darmstadt, Germany). A multiplex ELISA (metabolic panel Milliplex kit, EMD Merck Millipore Group) was used to determine fasting serum insulin, glucagon, leptin and monocyte chemoattractant protein-1 (MCP-1) in mice with LNCaP xenografts. Absorbance at 450 nm and 595 nm was determined using a FLUOstar Omega plate reader and software (BMG Labtech, Offenburg, Germany), with absorbance values interpolated using linear regression.

### Histological tissue analysis

Cryosections (6–10 μm thick, Leica CM1850 cryotome) were collected onto warm, charged Menzel Superfrost slides (Thermo Fisher), air dried for 1–2 hours and stored at −80 °C. Sections were fixed with ice-cold 100% acetone for 10 mins, followed by air-drying. White adipose tissue was processed and embedded in paraffin before sectioning (5 µM sections). One section from each specimen was stained with Mayer's haematoxylin and eosin (Sigma-Aldrich), and neutral lipids were stained in skeletal muscle and liver sections using oil-red-O stain (ORO; Sigma-Aldrich). Frozen sections were fixed in formalin, rinsed in 60% isopropanol, stained with ORO for 15 minutes, rinsed in 60% isopropanol, and mounted with coverslips using CC/Mount (Sigma-Aldrich). Stained sections were observed using an Olympus BX41/702 microscope (U-CMAD3) and the area of red, ORO-stained lipid (minimum n = 3 samples per group and n = 3 fields per section) quantified using the thresholding function in the ImageJ software (Research Services Branch, National Institute of Health, Washington, Maryland, USA)^53^. Adipocyte size (mean area of white adipose cells, minimum n = 3 samples per group and n = 3 fields per section) was quantified using the freehand area selection tool in ImageJ.

Immunohistochemistry was performed to investigate the expression of the inflammatory macrophage marker F4/80. After rehydration in a series of xylene and ethanol washes, and antigen retrieval (Carezyme Trypsin, Biocare Medical), tissue sections were incubated in 3% hydrogen peroxide for 10 min to block endogenous peroxidases. Sections were washed in phosphate buffered saline (PBS) followed by PBS with 0.05% Tween 20 (PBST) and a blocking step using 10% BSA in PBST. Rat anti-mouse F4/80 primary antibody (122602 Cell Signalling Technology, Massachusetts, USA) was diluted 1:50 in PBST with 10% BSA. Tissue sections were washed in PBST, incubated with HRP-polymer conjugates (SuperPicture, Thermo Fisher), and incubated with the chromagen diaminobenzidine (DAB) (Dako, Glostrup, Denmark), as per manufacturer's specifications. Slides were counterstained with Mayer's haematoxylin, dehydrated, and mounted with coverslips using D.P.X neutral mounting medium (Sigma-Aldrich). The number of F4/80 positive cells was quantified as a percent of the total number of cells in the field (n = 3 samples per group and n = 3 fields per section) using ImageJ software^53^.

### Statistics

Statistical analyses were performed using GraphPad Prism v6.01 (GraphPad Software, Inc., San Diego, CA, USA). Data were tested for normality using the Shapiro-Wilk test. Non-normally distributed data was analysed using non-parametric Kruskal-Wallis and Mann-Whitney U tests. Normally distributed data was analysed using parametric two-way ANOVA and Tukey's multiple comparison tests with *P* ≤ 0.05 considered to be statistically significant.

## Electronic supplementary material

Figure SI
