# Supplementary material for: Insights from engraftable immunodeficient mouse models of hyperinsulinaemia
Source: Sci Rep. 2017 Mar 28;7:491. doi: 10.1038/s41598-017-00443-x (PMC5428450; doi:10.1038/s41598-017-00443-x)
Supplement: Supplementary file 1 — Figure SI [file 41598_2017_443_MOESM1_ESM.pdf]

Supplementary Information: Insights from engraftable immunodeficient mouse models of hyperinsulinaemia

Michelle L. Maugham[1,2,3,5], Patrick B.Thomas[1,2,3,], Gabrielle J.Crisp[1,2,3,], Lisa K. Philp[1,2], Esha T. Shah[1,2,3], Adrian C. Herington[1,2], Chen Chen[4], Laura S. Gregory[5], Colleen C. Nelson[2], Inge Seim[1,2,3], Penny L. Jeffery[1,2,3*], Lisa K. Chopin[1,2,3*]

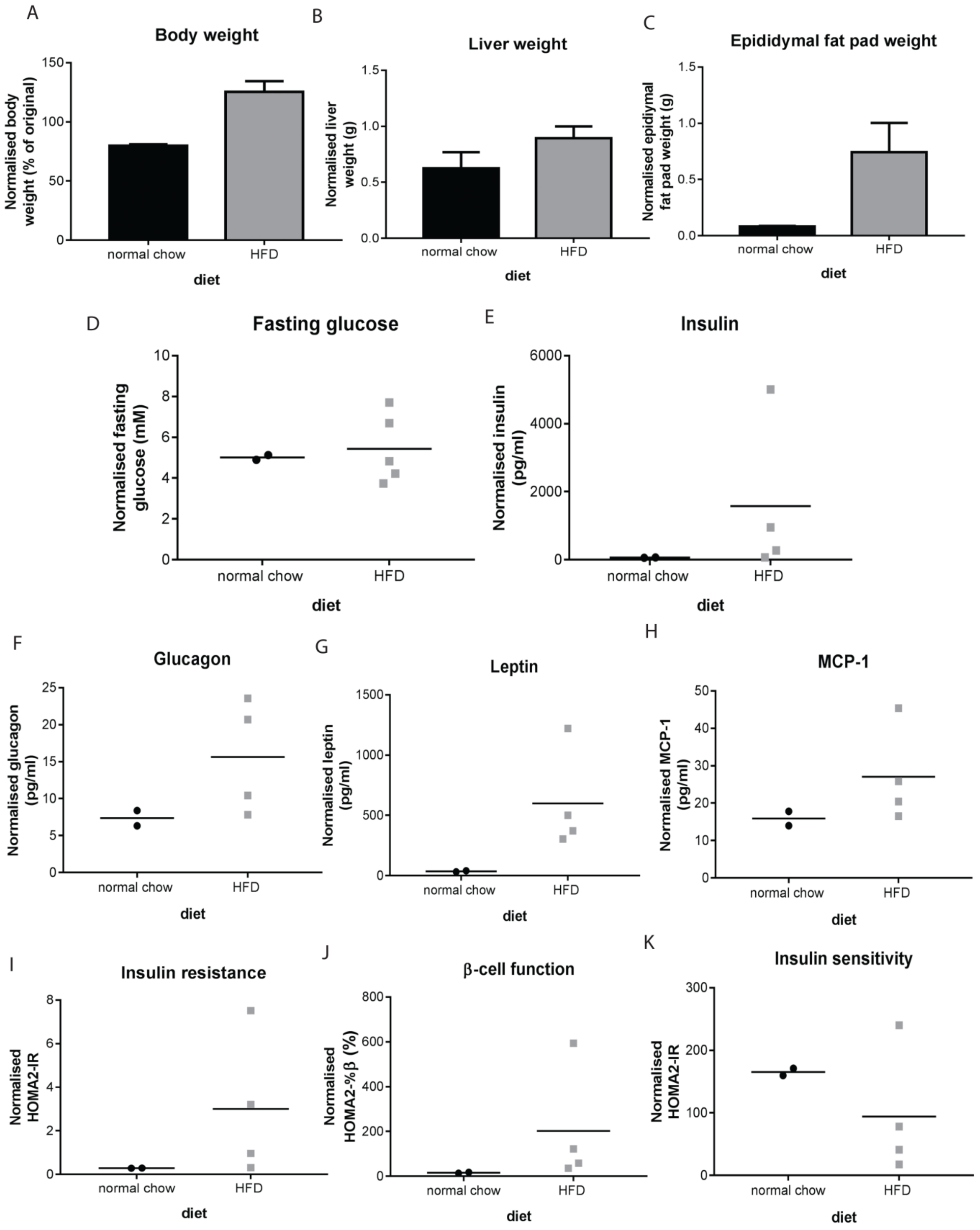

Figure S1. *Rag1*$^{-/-}$ mice with LNCaP xenografts fed a Western HFD (n=4-5) have more pronounced symptoms of metabolic syndrome compared to mice fed low-fat chow-fed (n=2). **(A)** Endpoint body weight (percent of original weight) of LNCaP xenograft implanted HFD-fed *Rag1*$^{-/-}$ mice compared to low-fat chow-fed *Rag1*$^{-/-}$ mice. **(B)** Endpoint liver weight in HFD and low-fat chow-fed *Rag1*$^{-/-}$ mice. **(C)** Epididymal fat pad weight in HFD-fed *Rag1*$^{-/-}$

mice compared to low-fat chow-fed *Rag1*$^{-/-}$ mice at endpoint. **(D)** Fasting serum glucose levels in LNCaP xenograft implanted HFD-fed *Rag1*$^{-/-}$ mice compared to low-fat chow-fed *Rag1*$^{-/-}$ mice. **(E)** Fasting serum insulin levels in *Rag1*$^{-/-}$ HFD and low-fat chow-fed mice. **(F)** Fasting serum glucagon levels are greater in HFD-fed *Rag1*$^{-/-}$ mice compared to low-fat chow-fed *Rag1*$^{-/-}$ mice at endpoint. **(G)** Fasting serum leptin levels in HFD-fed *Rag1*$^{-/-}$ mice compared to chow-fed *Rag1*$^{-/-}$ mice suggest dysregulation of energy balance in these HFD-fed mice. **(H)** Greater serum MCP-1 levels in HFD-fed *Rag1*$^{-/-}$ mice compared to chow-fed *Rag1*$^{-/-}$ mice indicate greater systemic inflammation indicative of metabolic dysregulation. **(I)** Insulin resistance (HOMA-IR) at endpoint is greater in HFD-fed mice compared to low-fat chow-fed mice. **(J)** Steady state β-cell function (HOMA% β) is higher in HFD-fed *Rag1*$^{-/-}$ mice compared to low-fat chow-fed *Rag1*$^{-/-}$ mice. **(K)** Insulin sensitivity (HOMA%S) is higher at endpoint in HFD-fed mice compared to low-fat chow-fed mice. All data have been normalised to time since weaning and is shown as mean + SEM. Statistically significant differences were determined by Kruskal-Wallis and Mann-Whitney tests. *P*= NS.
